# Computed tomography (CT) imaging evaluation of integrated traditional Chinese medicine cooperative therapy in treating acute cerebral infarction: A randomized controlled trial

**DOI:** 10.1097/MD.0000000000019998

**Published:** 2020-05-01

**Authors:** Ruijia Liu, Xudong Yu, Liping Zhang, Hong Zhang, Yuanyuan Gong, Kang Wu, Shuting Yan, Lianying Song

**Affiliations:** a Graduate School of Beijing University of Chinese Medicine; b The First Department of Neurology; c Department of Andrology; d Department of Radiology, Dongzhimen Hospital, Beijing University of Chinese Medicine, Beijing 100700, China.

**Keywords:** acute cerebral infarction, computed tomography, protocol, randomized controlled trial

## Abstract

**Introduction:**

Acute cerebral infarction is a clinically common cerebrovascular disease. Acute cerebral infarction is characterized by sudden onset, dangerous illness, high risk of death, and disability. Computed tomography perfusion imaging can detect abnormal brain tissue perfusion 30 minutes after the onset of cerebral ischemia, providing the earliest and most valuable information for clinical diagnosis and treatment. In recent years, the effect of traditional Chinese medicine on acute cerebral infarction has been remarkable.

**Methods/design:**

This study plan randomly divided eligible acute cerebral infarction patients into two groups. Patients in the control group will be treated with conventional Western medicine; patients in the intervention group will be treated with traditional Chinese medicine cooperative therapy on the basis of conventional Western medicine. The curative effects will be selected before treatment, 2 weeks after treatment, and 3 months follow-up. The changes in CT imaging evaluation, NIHSS score, and BI index of the two groups of patients will be observed.

**Discussion:**

We aim to provide higher evidence-based medical evidence for traditional Chinese medicine treatment of acute cerebral infarction. And clarify the application value of computed tomography perfusion imaging in the diagnosis and efficacy evaluation of acute cerebral infarction.

**Trial registration:**

ClinicalTrials.gov, ChiCTR2000030230, Registered on 03 March 2020.

## 1 Introduction

Acute cerebral infarction (ACI) is a clinically common cerebrovascular disease. ACI is characterized by sudden onset, dangerous illness, high risk of death, and disability.^[1]^ In recent years, the incidence of cerebrovascular disease has gradually increased. According to the WHO Global Disease Data Report, cerebral infarction is the third leading cause of death among residents worldwide, and it is also a major cause of disability.^[2,3]^ In recent years, the increase in the number of patients with acute cerebral infarction may be related to life, work, psychological stress, and incontinent diet. In addition, atherosclerosis and cardiogenic embolism are the two most common causes of cerebral infarction.^[4]^ The former can cause narrowing of the arterial lumen, leading to brain tissue necrosis. Another common cause of cerebral infarction is cardiogenic embolism. Most embolic emboli will stagnate on the head and cause ACI.

In terms of treatment, Western medicine has a greater limitation in the treatment of cerebral infarction.^[5]^ Western medicine treatment of cerebral infarction is mainly based on thrombolysis, antiplatelet aggregation drugs, and scavenging oxygen free radicals. The use of anti-platelet aggregation drugs is prone to bleeding, gastrointestinal reactions, liver and kidney damage, and is not suitable for patients to take for a long time. In addition, for the treatment of ACI, clinically, intravenous thrombolysis is often used to treat the disease.^[6]^ However, most patients have missed the thrombolytic time window when they were admitted to the hospital, or are unable to take thrombolytic therapy because of the uncertain onset time. With the continuous development of imaging diagnosis technology and equipment, the current ultra-early cerebral infarction diagnosis technology solutions available for clinical use are no longer limited to conventional computed tomography (CT) plain scans, and more and more new technical methods are being developed and utilized photon emission tomography, positron emission tomography, etc.^[7]^ However, the above-mentioned new diagnostic technology has not been popularized in primary medical institutions, and the medical burden is too heavy and the operation time is too long. The inability to complete the assessment of cerebral blood flow in emergency patients in a short time has become a difficult bottleneck for the development of this disease.^[8,9]^ Studies have found that CT perfusion imaging (CTPI) can detect abnormal brain tissue perfusion 30 minutes after the onset of cerebral ischemia, providing the earliest and most valuable information for clinical diagnosis and treatment. In recent years, the effect of traditional Chinese medicine (TCM) on ACI has been remarkable. Therefore, this study intends to use a clinical randomized controlled study to explore the exact effect of TCM in the treatment of acute cerebral infarction, and use CTPI imaging methods to evaluate the effect.

## 2 Methods/design

### 2.1 Study design and settings

**Figure 1:**
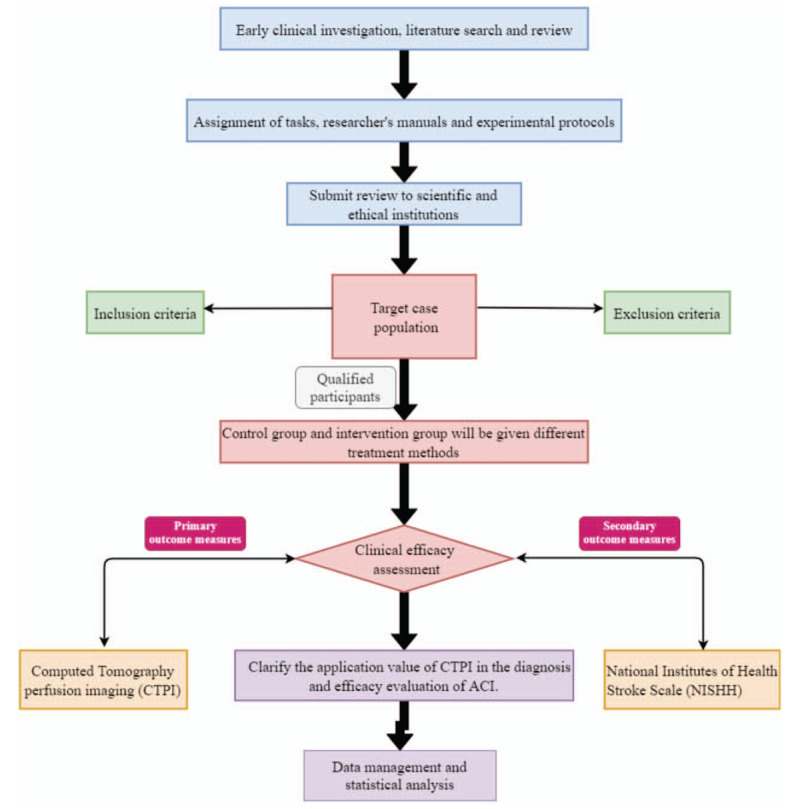
Study design flow chart.

A brief flowchart of the entire study is shown in Figure 1. We will perform a 2-group, randomized, single-blind, placebo-controlled, and multi-center trial that will evaluate the efficacy and safety of TCM cooperative therapy for patients with ACI. This study will use a completely random grouping and parallel control observation design method. We will ensure the balance of the baseline data of the two groups through a sufficient sample size and a completely randomized grouping method. The protocol includes elements recommended in the Standard Protocol Items: Recommendations for Interventional Trials checklist (Additional file 1).

### 2.2 Participants

All cases will be included in patients with acute stage of cerebral infarction admitted to the Department of Neurology, Dongzhimen Hospital. CTPI examination of the head will be completed. They will be randomly divided into two groups, the intervention group and the control group.

#### 2.2.1 Diagnostic criteria

The Western medicine diagnosis criteria of ACI will refer to the “*Guidelines for the Diagnosis and Treatment of Chinese Acute Ischemic Stroke*” revised by the Chinese Medical Association Neurology Branch in 2018. The diagnostic criteria are as follows:

(1) The patient has an acute onset.
(2) Focal neurological deficits (numbness/weakness on one side of the limb, with or without speech impairment, etc.).
(3) Responsible lesions or symptoms/signs appear on imaging for more than 24 hours.
(4) Exclude non-vascular causes.
(5) Brain CT/MRI excludes cerebral hemorrhage.

The diagnostic criteria of TCM will refer to the “*Stroke Diagnosis and Efficacy Evaluation Standards”*. The specific summary is as follows: (1) the main symptoms: ① hemiplegia; ② skewed tongue; ③ speech is astringent or unable to speak; ④ sensory function decline, disappearance, numbness. (2) Secondary symptoms: ① dizziness; ② upset and irritable; ③ stiff limbs; ④ sputum is sticky; ⑤ drinking water will cause coughing; ⑥ ataxia. Have more than 2 main symptoms, or 1 main symptom and 2 secondary symptoms, combined with the characteristics of acute onset, inducement, aura symptoms and imaging examination can confirm the diagnosis.

#### 2.2.2 Inclusion criteria

(1) Those who meet the ACI diagnostic criteria for Western medicine;
(2) Those who meet the diagnostic criteria for stroke in Chinese medicine;
(3) The time from onset to admission for treatment is within 72 hours;
(4) The National Institutes of Health Stroke Scale (NIHSS) score is ≥6 points and ≤22 points;
(5) Patients with first onset of ACI or patients with recurrent stroke have fully recovered before the onset of this disease;
(6) Those who voluntarily accept the clinical observation of this project and sign the informed consent;

#### 2.2.3 Exclusion criteria

Patients will be excluded if they meet the following criteria:

(1) Large-scale cerebral infarction, the vital signs are not stable;
(2) Transient ischemic attack or cerebral hemorrhage;
(3) With severe heart, liver, lung, kidney, and hematopoietic system diseases;
(4) Pregnancy or lactation period;
(5) Mental abnormalities and the patient who cannot cooperate.

#### 2.2.4 Case rejection or shedding criteria

(1) Allergic reactions or serious adverse events occur.
(2) In the course of clinical observation, the patient developed other diseases and did not meet the inclusion criteria.
(3) Subjects have poor compliance (compliance with test drugs <80% or> 120%), or change medications automatically.
(4) Cases in which blindness is disrupted midway for various reasons.
(5) Patients and their families resolutely terminate the experiment.

#### 2.2.5 Suspension criteria

**Figure 2:**
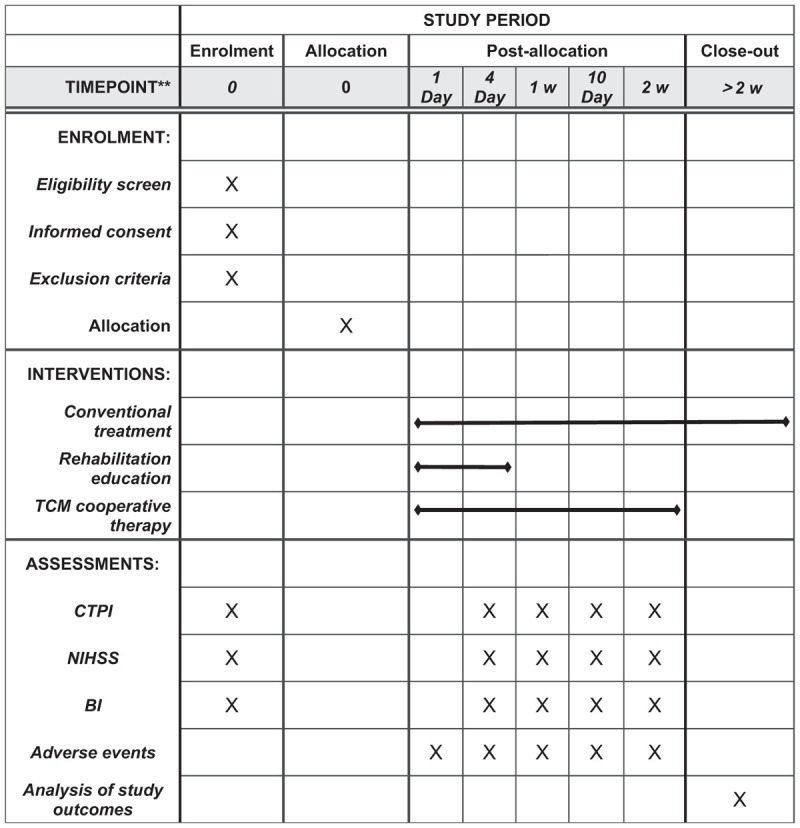
SPIRIT figure for the schedule of enrollment, interventions, and assessments. CTPI = computed tomography perfusion imaging, NIHSS = National Institutes of Health Stroke Scale, SPIRIT = Standard Protocol Items: Recommendations for Interventional Trials.

(1) In the treatment of this study, the patient was unexpectedly pregnant or had serious adverse reactions, and it was difficult to continue to receive treatment.
(2) During the implementation of this study, there was an obvious exacerbation or complications.(Fig. 2)

### 2.3 Interventions

#### 2.3.1 Different interventions given in two groups

The control group will be given basic Western medicine treatment. The course of treatment was 2 weeks, and the effect was observed after 2 weeks. Followed up to 3 months after onset. We will refer to the “Chinese Guidelines for the Diagnosis and Treatment of Acute Ischemic Stroke 2018 Edition” for the treatment of cerebral infarction formulated by the Chinese Medical Association Neurology Branch. Specific plans are: aspirin 100 mg, clopidogrel hydrogen sulfate 75 mg, orally once a day before bedtime; and routine nutritional nerves, lowering fat density, and improving microcirculation treatment for patients with acute stage of cerebral infarction; related to other diseases Specialist treatment, but it should be noted whether the medication affects the effect of the test drug and recorded in detail. The intervention group will be given TCM cooperative therapy. In addition, the two groups will be given basic Western medicine treatment at the same time. If there are hypertension, coronary heart disease, diabetes, infection, electrolyte disturbance and other symptoms, we will give routine treatment and symptomatic treatment.

#### 2.3.2 CT volume cerebral perfusion imaging

All patients will undergo conventional CT plain scan and spiral CT brain perfusion imaging examination. We will use Siemens dual-source CT *(Device model: Somatom Definition Flash, device number: 73396)* for operation. After the conventional craniocerebral CT scan, the DynMulti4D scan mode was used for VPCT scan. A double-barreled high-pressure syringe will be used to inject an iohexol 50 ml through an anterior cubital vein 18–20G at a flow rate of 5 ml/s. 40 ml of normal saline will be injected at the same flow rate; continuous dynamic scanning will be started 21 times 5 s after the contrast injection, and the scanning time is 35.54 s. Scanning parameters: tube voltage 80 kV, tube current 120 mAs, scanning range 100 mm, tube rotation time 0.28 s/rev, collimator width 128 × 0.6 mm. Automatic reconstruction of 5 mm and 1 mm axial image, 1 mm The axial image is automatically transferred to the workstation. Image evaluation: The scanned images need to be processed by software in order to obtain information such as cerebral flow and cerebral blood volume on the lesion side, contralateral side, and other parts.

#### 2.3.3 CT Image post-processing

The 5 mm perfusion image will be post-processed with the Neuro PCT software package on the scanning table. The software automatically generates three-dimensional VPCT parameter maps: cerebral blood flow (CBF), cerebral blood volume (CBV), mean transit time (MTT), and time to drain (TTD). The 1 mm layer CTA data will be post-processed on the workstation using Inspace software to obtain 21 periods of dynamic CTA images (4D-CTA). Post-processing methods include VRT, MIP, and MPR.

### 2.4 Outcome measures

#### 2.4.1 Primary outcome measures

The primary outcome measure will be evaluated using CT imaging parameters. Brain CTPI parameters collection method: Siemens dual-source CT *(Device model: Somatom Definition Flash, device number: 73396),* CTPI imaging examination of patients with unknown onset time. The opposing areas of the cerebral hemispheres on both sides are measured to quickly determine if there is a reversible ischemic zone. Using a high-pressure syringe, a nonionic contrast agent is injected into the patient's anterior cubital vein. Then, a single layer of continuous dynamic scanning is performed on the selected layer. The resulting image is processed by Siemens CTPI software package for subsequent processing. We will use the midline of the brain as the mirror to select the area of interest, and measure the lesion area and the corresponding area on the opposite side symmetrically. We will use this method to obtain cerebral blood flow (CBF), cerebral blood volume (CBV), and mean transit time (MTT) at different levels on both sides of the brain.

#### 2.4.2 Secondary outcome measures

The National Institutes of Health Stroke Scale (NIHSS) will be used to assess the neurological function of patients with ACI, and the Barthel Index (BI) score was used to assess the ability of daily living.

#### 2.4.3 Safety observation measures

(1) General physical examination items, such as body temperature, heart rate, respiration, blood pressure, and blood sugar.
(2) Possible adverse events and the incidence of adverse events.

### 2.5 CT Imaging evaluation method

The CTPI images collected in all cases will be evaluated blindly by 2 physicians who have more than one diagnosis. After jointly determining the perfusion abnormal area and reaching an agreement, the region of interest (ROI) is manually drawn. The ROI selects the center of the lesion, and simultaneously draws the contralateral mirror area on the centerline symmetry axis. When the symmetry area is an abnormal perfusion, the normal area of the contralateral same-layer perfusion parameter map is selected as the contralateral mirror area. CBV, TTD, and MTT values. Infarct focus was based on review of MRI DWI high signal area. The degree of stenosis of the responsible vessels was also evaluated.

### 2.6 Randomization and blinding

The random method will use the closed envelope method. We will refer to the random number table to generate random arrangements for the intervention and control groups. The corresponding treatment serial number is sealed and placed in a light-tight envelope to form a random letter. Patients will be randomly assigned to the intervention group or the control group in the order of the included cases. In addition, at the time of drug distribution, all tested drugs must be accompanied by a corresponding numbered emergency letter before distribution. Medicines will be distributed according to the group and medicine number assigned to each patient's visit number. The number of the drug must remain the same during clinical observations.

### 2.7 Statistical analysis

SPSS for windows 24.0 statistical analysis software will be used for calculation, and normality test and homogeneity test of variance will be performed on each group of data. For measurement data in which the data conforms to the normal distribution, we will use the mean ± standard deviation. For nonnormally distributed measurement data, the median ± quartile interval is used. General data comparison between the two groups using independent sample T test. Comparisons before and after treatment will be performed using t test for paired data. One-way analysis of variance will be used for comparison between groups. X^2^ test will be used for count data, and nonparametric rank sum test will be used for rank data. All statistical tests are two-sided. *P* < .05 indicates a significant difference.

### 2.8 Data management

Information obtained from the evaluation of each participant will be recorded on a paper print-out. The information will then be handwritten on a paper document case report form and entered into an Excel file for future statistical analyses. In accordance with the Personal Information Protection Act, the names of all participants will not be disclosed, and a unique identifier number given during the trial will be used to identify participants. All of the participants will be informed that the clinical data obtained in the trial will be stored in a computer and will be handled with confidentiality. The participants’ written consent will be stored by the principal investigator.

### 2.9 Ethics

This study will be approved by the Ethics Committee of Dongzhimen Hospital Affiliated to Beijing University of Chinese Medicine. The study will be conducted under the Declaration of Helsinki principles, as well as following the norms of good clinical practice. Recruitment of patients has not started in this study. The study plan will be submitted to the ethics committee of the Ethics Committee of Dongzhimen Hospital Affiliated to Beijing University of Chinese Medicine for review. We will not start recruiting participants without the consent of the ethics committee.

## 3 Discussion

The early diagnosis of ACI is of great significance to improve the prognosis of patients and save patients’ lives.^[10]^ Because only when the condition is found and diagnosed early in the onset of cerebral infarction (within 6 hours) can subsequent treatment such as intravenous thrombolysis be advanced. In this way, we can grasp the optimal timing of thrombolysis and reduce the degree of brain tissue damage.^[11,12]^ In addition, it is clinically recognized that changes in cerebral blood flow can feed back pathological changes in human brain tissue.^[13]^ For example, cerebral hemodynamic changes in patients with cerebral infarction have obvious characteristics. It is mainly manifested in local neurochemical changes in the early stages of onset.^[14]^ In order to ensure the local normal blood supply, the cerebral blood vessels will inevitably show a state of compensatory expansion. Corresponding resistance to cerebrovascular circulation will be significantly reduced. Imaging showed increased cerebral blood flow and prolonged mean transit time of contrast medium.^[15]^ When the vasodilation reaches the upper limit, cerebral blood flow will decrease. Once the state persists for more than 6 hours, it means that the blood supply to the brain is greatly reduced. At this time, cerebrovascular dilatation reaches a decompensated state, which in turn causes irreversible damage. Some scholars have stated in research that comprehensively grasping the patient's brain imaging diagnostic information in the early stages of onset will help physicians to correctly judge the condition such as whether the central neuron is necrotic, whether there are penumbras in the surrounding area, and the area of the penumbra area is geometric.^[16,17]^ Take first aid measures symptomatically. It is not difficult to see that clarifying the lesions and their changes in cerebral blood flow is very important to reverse the outcome of brain tissue. And how to obtain more comprehensive and valuable information about blood flow in living cells has always been the focus of investigation in imaging medicine.

ACI belongs to the category of “stroke” in TCM.^[18]^ Mostly caused by overwork, poor diet and emotional damage. Studies have shown that traditional Chinese herbal medicines has the effects of increasing high-density lipoprotein, reducing low-density lipoprotein, regulating blood lipids, stabilizing plaque, and protecting vascular endothelium.^[19–21]^ In addition, in the early stages of ACI, Chinese herbal medicines can remove free radicals and reduce damage caused by free radicals.^[22,23]^ This can reduce blood viscosity, thereby improving the oxygen carrying capacity of red blood cells and preventing brain tissue damage.^[24,25]^ In summary, this study plans to use TCM cooperative therapy to treat ACI and use CTPI imaging to evaluate the efficacy. We aim to provide higher evidence-based medical evidence for TCM treatment of ACI. And clarify the application value of CTPI in the diagnosis and efficacy evaluation of ACI.

### 3.1 Trial status

At the time of manuscript submission, recruitment for the study is not yet started.

## Acknowledgments

The authors would like to thank all the trial participants. The authors are grateful for the support for this study: trial coordinating team, nurses, and research departments.

## Author contributions

RJL, XDY, and KW designed the study protocol and drafted the manuscript. LYS and YYG reviewed the study protocol and drafted the manuscript. STY and HZ are responsible for the statistical design and analysis as trial statistician. All authors carefully read and approved the final version of the manuscript. LPZ participated in the design and coordination of the study. All authors read and approved the final manuscript.

Lianying Song orcid: 0000-0002-9365-6046.
